# Altered expression of costimulatory molecules in dementias

**DOI:** 10.1007/s00406-021-01297-1

**Published:** 2021-08-24

**Authors:** Stefan Busse, Franz von Hoff, Enrico Michler, Roland Hartig, Bernhard Bogerts, Mandy Busse

**Affiliations:** 1 Department of Psychiatry, University of Magdeburg, Leipziger Str. 44, 39120 Magdeburg, Germany; 2 Institute of Immunology, University of Magdeburg, Magdeburg, Germany; 3 Experimental Obstetrics and Gynecology, Medical Faculty, Otto-von-Guericke University, Magdeburg, Germany

**Keywords:** Alzheimer’s disease, Vascular dementia, Frontotemporal dementia, T cells, Costimulation

## Abstract

Although the expression of co-stimulatory molecules plays an important role in the immune system, only little is known about their regulation in dementias. Therefore, we determined the expression of CD28, ICOS (CD278) and CTLA-4 (CD152) by CD4 + and CD8 + T cells in the peripheral blood of patients with mild cognitive impairment (MCI; *N* = 19), Alzheimer’s disease (AD; *N* = 51), vascular dementia (VD; *N* = 21) and frontotemporal dementia (FTD; *N* = 6) at the point in time of diagnosis compared to 19 non-demented elderly persons. The expression of CD28 and ICOS by CD4 + and CD8 + T cells was not changed in AD, FTD or VD patients. The expression of the negative regulator CTLA-4 was increased by CD4 + T cells from AD and FTD patients and by CD8 + T cells from VD patients. The classification of the AD patients according to the severity of the disorder showed stage-dependent alterations of CD28, ICOS and CTLA-4 expression. In AD patients, the correlation analysis showed an association between the decline in CD28 + T cells and the increase in CTLA-4 + T cells with cognitive decline, measured by the mini-mental state examination (MMSE), tau proteins and Amyloid-β, important AD biomarkers in cerebrospinal fluid (CSF). In FTD patients, a positive association between Q Albumin, a marker for blood-CSF-barrier function, and CD28 and a negative correlation between Q Albumin and ICOS expression were determined. Our data suggest a dysregulated balance between the expression of negative and positive co-stimulatory molecules by T cells in AD patients, which might contribute to chronic inflammation observed in dementia.

**Supplementary Information:**

The online version contains supplementary material available at 10.1007/s00406-021-01297-1.

## Introduction

In our aging population, the prevalence of dementia is increasing. In 2010, approximately 35.6 million people worldwide suffered from dementia, a number which is expected to triple by the year 2050. Each year, 7.7 million people were newly diagnosed with dementia worldwide. The most prevalent cause of dementia is Alzheimer’s disease (AD). Besides, vascular dementia (VD) and frontotemporal dementia (FTD) contribute to the loss of cognitive functions and behavioural changes in elderly people [1–4]. In AD, Amyloid-β1-42, and tau proteins are established core cerebrospinal biomarkers [3]. FTD is a heterogeneous neurodegenerative disorder with some symptoms overlap with AD [5]. Some FTD forms are tauopathies, they are caused by a loss of tau protein expression [6]. VD has multifactorial causes, but vascular components such as atherosclerosis, (micro)infarcts and amyloid angiopathy play an important role [4].

Aging is also associated with a progressive decline in immune competence of T and B lymphocytes. T cell senescence is characterized e.g. by the loss of co-stimulatory receptors such as CD28 and impairment of TCR signaling, leading to defects in classical T cell functions [7, 8]. Moreover, the term “inflamm-aging” was created to describe the presence of a chronic state of inflammation in many age-associated diseases. It is believed that inflammatory immune responses [9–11] and a dysfunction of the blood-cerebrospinal fluid (CSF)-barrier (B-CSF-B) [12, 13] are relevant in AD, FTD and VD.

During conventional T cell activation, the interaction of the T cell receptor (TCR) with MHC class I or class II-peptide complexes initiates the cascade of T cell activation. However, an important secondary co-stimulatory signal must be delivered to promote cellular proliferation and survival following T cell activation [14]. This “second signal” is provided by the co-stimulatory molecule CD28 [15]. CD28 is a homodimeric cell surface receptor of the immunoglobulin superfamily to which also belong the co-receptor molecules cytotoxic T-lymphocyte antigen-4 (CTLA-4; CD152) and inducible T cell co-stimulator (ICOS) [16–18].

While CD28 is expressed on the majority of human CD4 + T cells but only about half of circulating CD8 + T cells, CTLA-4 is constitutively expressed on regulatory T (Treg) cells and upregulated by activated conventional T-cells and also ICOS is rapidly induced following T cell activation [16, 19]. CD28 mediated co-stimulation controls T cell responses by providing means of preventing unwanted (anti-self) and triggering wanted (antimicrobial) immunity while CTLA-4 ligation serves to mitigate this process [18].

Previously we have shown alterations in the adaptive immune system in patients suffering from AD, VD and FTD, in particular AD associated changes in the T cell naïve/ memory subpopulations [20, 21]. Therefore, we wanted to determine whether also the expression of the T cell co-stimulatory markers CD28, ICOS and CTLA-4 was affected by dementia.

## Materials and methods

### Study cohort

The study was performed in accordance with German laws, the Declaration of Helsinki and the guidelines of the local institutional review board. Written consent was obtained from all patients and healthy volunteers. 10 ml blood tubes were collected from 19 MCI patients (14 female, 5 male; mean age 76.74 years), 51 AD patients (32 female, 19 male; mean age 80.46 years), 21 VD patients (13 female, 8 male; mean age 78.14 years) and 6 FTD patients (2 female, 4 male; mean age 74.33 years). Diagnosis was performed by psychiatrists, neurologists and psychologists according to DSM-IV criteria, based on the results obtained by magnetic resonance imaging (MRI) of the brain or cerebral computer tomography (CT), cerebrospinal fluid (CSF) analysis, mini-mental state evaluation (MMSE) and electroencephalography (EEG). CSF analysis, including levels of Amyloid-β1-40, Amyloid-β1-42, total tau, phospho-tau and Q-Albumin, was performed at the institute of clinical chemistry and pathobiochemistry of our university hospital. The levels of Amyloid-β1-40, Amyloid-β1-42, total tau, phospho-tau (^181^P) were determined by using the INNOTEST^®^ immunoassays, solid-phase enzyme immunoassays (Fujirebio, Gent, Belgium). Q Albumin was measured on IMMAGE^®^ 800 Protein Chemistry Analyzer (Beckman Coulter). Clinicians had access to detailed clinical files, including the medical histories by proxy and referral letters from the general practitioners. The stages of AD were divided according to the results obtained from MMSE: MMSE scores 24–20: mild AD; MMSE 19–10: moderate AD; MMSE below 10: severe AD. 19 elderly non-demented persons (13 female, 6 male; mean age 71.22 years) donated blood as controls. The demographic data of our study cohort, including MMSE values and Q Albumin values, are shown in Table 1. From all patients and control individuals, routine blood analyses (including differential blood cell count, levels of C-reactive protein, glucose, lipids, liver enzymes and thyroid hormones) were performed. Abnormal routine blood values (e.g. enhanced CRP or abnormal immune cell counts), a history of severe immune diseases, immunomodulating treatment, cancer, chronic terminal disease, substance abuse or severe trauma were exclusion criteria of the study.

**Table 1:** Demographic data of the study cohort

Characteristics	Controls	MCI	AD				VD	FTD
			Total	Mild	Moderate	Severe		
Total (*n*)	19	19	51	13	26	12	21	6
Age (years; mean)	71.2	76.7	80.5	81.5	80.1	80.4	78.1	74.3
Gender (female/male)	13/6	14/5	32/19	9/4	14/12	9/3	13/8	2/4
MMSE (mean)	28.1	26.3	15.3	21.5	15.5	7.4	16.7	20.7
Q Albumin (mean)		8.2	10.1	8.6	9.6	12.8	10.3	7.9

CSF diagnostics	AD		
	Mild	Moderate	Severe
MMSE (mean)	21.54	15.52	7.36
Q albumin (mean)	8.60	9.59	12.75
Total tau (pg/ml)	628.3	701.4	787.7
Ptau (pg/ml)	106.70	91.45	82.22
Amyloid-β1-40 (pg/ml)	10,690	9338	7514
Amyloid-β1-42 (pg/ml)	519.0	540.0	448.9

The demographic data of the study cohort including age, gender, MMSE values and Q albumin are shown in Table 1a. AD specific markers total and phospho-tau, Amyloid-β 1–40 and 1–42 obtained in cerebrospinal fluid (CSF) from patients with mild, moderate and severe AD were listed in Table 1b

### Flow cytometry analysis

50 µl EDTA-blood per tube were incubated with the primary antibodies (Abs) at room temperature (RT) for 20 min. in the dark: Fluorescein (FITC) anti-human CD4 (M-T466), VioGreen anti-human CD8 (BW135/80) VioBlue anti-human ICOS (REA192), Phycoerythrin (PE) anti-human CD28 (15E8) (all Abs from Miltenyi Biotech, Bergisch Gladbach, Germany). Afterwards, 450 µl erythrocyte lysis buffer (eBioscience, Frankfurt, Germany) was added to the tube and incubated at RT for 30 min. in the dark.

For intracellular staining of CTLA-4, the cells were washed twice using FACS buffer and the pellet was incubated with 100 µl Fix/ Perm buffer (BD Biosciences) at RT for 30 min. in the dark. Then the cells were washed twice with perm buffer and incubated with allophycocyanin (APC) anti-human CTLA-4 (BNI-3; Miltenyi Biotech, Bergisch Gladbach, Germany) at RT for 30 min. in the dark. After two washing steps, data were collected on a FACS flow cytometer (LSR Fortessa, BD Biosciences, Mountain View, CA, USA) and analyzed using FACS DIVA software 6.1.3 (BD Biosciences, Mountain View, CA, USA) and FlowJo software (Treestar Inc., Ashland, OR, USA). The data were analyzed using biexponential transformation function for complete data visualization.

### Determination of B-CSF-B integrity

The blood-cerebrospinal fluid (CSF)-barrier (B-CSF-B) function was calculated by quotient (Q) albumin. Therefore, the concentration of albumin was analyzed in CSF and in parallel also in serum. Q albumin was calculated by dividing the detected concentration of albumin in CSF by that detected in serum (result: Q albumin). Since normal aging is associated with a decline in B-CSF-B functions, we calculated an age-dependent reference value of B-CSF-B in order to differentiate modifications of B-CSF-B induced by aging from disorder-related alterations.

### Statistical analysis

Statistical analysis was performed using GraphPad Prism 7. Diagnostic group differences were calculated by Kruskal–Wallis test, followed by Mann–Whitney test and Dunn’s multiple comparison test. Shapiro–Wilk normality tests were performed to analyze normal distribution. Correlation analyses were performed according to Pearson. Significance was defined as *p* < 0.05.

## Results

### Altered expression of CTLA-4 in dementias

**Fig. 1:**
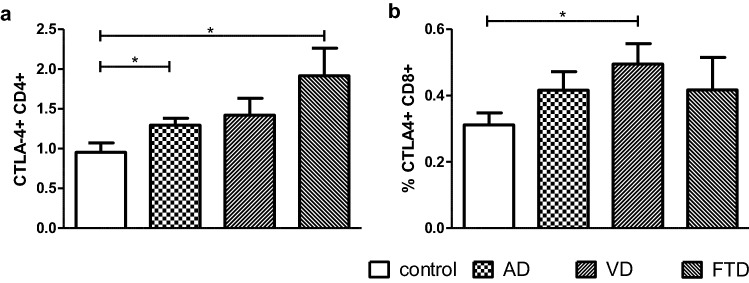
Expression of CTLA-4 in AD, VD and FTD. The expression of the negative regulator CTLA-4 on CD4 + T cells (**a**) and CD8 + T cells (**b**) from non-demented controls and patients with AD, VD and FTD at diagnosis were shown as percentage. *p < 0.05

Using flow cytometry of peripheral whole blood, we analyzed the frequency of CD4 + and CD8 + T cells and the expression of CD28, CTLA-4 and ICOS in patients suffering from AD, VD and FTD and compared them to non-demented controls. We detected in AD and in VD, but not in FTD a reduction of the percentage of CD4 + and CD8 + T cells (Suppl. Table 1). No diagnosis-dependent changes regarding the expression of CD28 and ICOS by CD4 + and CD8 + T cells were observed (Suppl. Fig. 1). However, the expression of CTLA-4 on CD4 + T cells among CD4 + T cells increased from 1% in the control group to 1.3% in AD (*p* = 0.0282) and 1.9% in FTD (*p* = 0.0118; Fig. 1a). Within the CD8 + T cell population of VD patients, on average 0.5% CD8 + T cell co-expressed CTLA-4 compared to 0.3% CTLA-4 + CD8 + T cells in non-demented individuals (*p* = 0.0271; Fig. 1b). Despite trends, no correlations were determined between the age of patients and control individuals and the frequency of CD4 T cells expressing CD28 (*p* = 0.0988), CTLA-4 (*p* = 0.3127) or ICOS (*p* = 0.4833) and between age and the number of CD8 + T cells expressing CD28 (*p* = 0.9272), CTLA-4 (*p* = 0.0644) or ICOS (*p* = 0.0685; data not shown).

### Influence of the stage of AD upon expression of costimulatory molecules

**Fig. 2:**
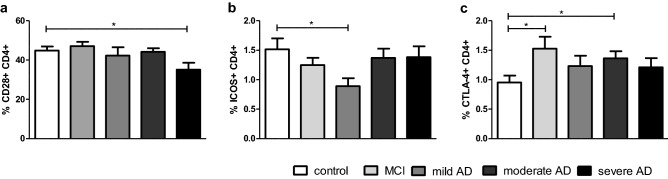
Expression of CTLA-4 in MCI and in stages of AD. The expression of CD28 (**a**), ICOS (**b**) and CTLA-4 (**c**) on CD4 + T lymphocytes were determined in patients suffering from MCI and AD patients grouped according to the severity of symptoms in mild, moderate and severe and compared to non-demented volunteers. *p < 0.05

Patients suffering from MCI show an increased risk for the development of dementia. Traditionally, MCI is regarded as the typical prodromal stages of dementia due to AD, but nonamnestic MCI might also lead to FTD or LBD [22]. AD can be subdivided according to the severity of symptoms into mild, moderate and severe AD. In several stages of AD, but not in MCI, we found diminished CD4 + and CD8 + T cell frequencies (Suppl. Table 1). Although the expression of CD28 and ICOS was unaltered in AD patients at the point in time of diagnosis, a stage-dependent analysis showed that the expression of CD28 on CD4 + T cells decreased from mean 44.8% in non-demented controls to 35.2% in patients with severe AD (*p* = 0.0270; Fig. 2a). The proportion of ICOS + CD4 + T cells declined from mean 1.5% in control persons compared to 0.9% ICOS + CD4 + T cells in the group of patients suffering from mild AD (*p* = 0.0396; Fig. 2b).

In MCI patients (mean 1.5% CTLA-4 + CD4 + /CD4 + ; *p* = 0.0392; Fig. 2c) and in patients with moderate AD (mean 1.4% CTLA-4 + CD4 + /CD4 + ; *p* = 0.0187), the expression of CTLA-4 by CD4 + T cells was increased compared to non-demented controls (average 1% CTLA-4 + CD4 + /CD4 +). No alterations of CD28, ICOS or CTLA-4 were detected in CD8 + T cells.

### Correlation between co-stimulatory molecules and AD-associated alterations in CSF

Alterations of phospho-tau (p-tau) and Amyloid-β 1–42 within CSF belong to the core features of AD and the determination of these values are part of the routine dementia diagnostics. Correlation analyses showed an association between the frequency of CD28 + CD8 + T cells and p-tau (*p* = 0.004), total tau (*p* = 0.0292), Amyloid-β 1–40 (*p* < 0.0001), and the Amyloid-β ratio (Amyloid-β 1–42/1–40; *p* = 0.0338) and a positive correlation between ICOS + CD8 + T cells and total tau (*p* = 0.0489) in AD patients (Table 2a).

**Table 2:** Correlation between biomarkers in CSF and peripheral lymphocyte populations in AD

AD (total)	CD28 + CD8 +				ICOS + CD8 +
	Phospho-tau	Total tau	Amyloid-β1-40	Amyloid-β ratio	Total tau
Pearson *r*	− 0.5178	− 0.4125	− 0.6642	0.3954	0.3755
*P* value (two- tailed)	0.004	0.0292	< 0.0001	0.0338	0.0489
*P* value summary	**	*	***	*	*
*R* square	0.2681	0.1701	0.4412	0.1563	0.141

Protein	Total tau		Phospho-tau		
AD stage	Mild AD	Moderate AD	Moderate AD		Severe AD
Cell population	CD28 + CD4 +	CTLA-4 + CD8 +	CD28 + CD8 +	CTLA-4 + CD8 +	CTLA-4 + CD8 +
Pearson *r*	− 0.9416	0.7055	− 0.6408	0.6581	0.9098
*P* value (two-tailed)	0.0005	0.0153	0.0336	0.0277	0.0017
*P* value summary	***	*	*	*	**
*R* square	0.8866	0.4978	0.4107	0.4331	0.8278

Protein	Amyloid-β1-40				Amyloid-β1-42		Amyloid-β ratio
AD stage	Mild AD			Moderate AD	Mild AD		Moderate AD
Cell population	CTLA-4 + CD4 +	ICOS + CD4 +	CD28 + CD8 +	CD28 + CD8 +	CTLA-4 + CD4 +	ICOS + CD4 +	CD28 + CD4 +
Pearson *r*	− 0.699	− 0.752	− 0.7644	− 0.6703	− 0.7341	− 0.7096	0.6944
*P* value (two-tailed)	0.0362	0.0194	0.0165	0.024	0.0243	0.0323	0.0177
*P* value summary	*	*	*	*	*	*	*
*R* square	0.4886	0.5655	0.5843	0.4493	0.5389	0.5035	0.4822

Table 2a illustrates the correlation between CD28 + CD8 + T cells and phospho-tau, total tau, Amyloid-β 1–40 and the Amyloid-β ratio and the correlation between ICOS + CD8 + T cells and total tau in AD patients

Table 2b demonstrates the correlation between total tau and phospho-tau and CD28 + CD4 + , CTLA-4 + CD8 + and CD28 + CD8 + T lymphocytes in mild, moderate ad severe AD

In Table 2c, the correlation between Amyloid-β 1–40, Amyloid-β 1–42 and Amyloid-β ratio and the percentage of CTLA-4 + CD4 + , ICOS + CD4 + and CD28 + CD8 + T cells in peripheral blood of patients with mild and moderate AD is disclosed

A stage-dependent analysis revealed a negative correlation between CD28 + CD4 + T cells and total tau (*p* = 0.0005; Table 2b), between CTLA-4 + CD4 + T cells and Amyloid-β 1–40 (*p* = 0.0362) and Amyloid-β 1–42 (*p* = 0.0243; Table 2c) and between ICOS + CD4 + T cells and Amyloid-β 1–40 (*p* = 0.0194) and Amyloid-β 1–42 (*p* = 0.0323) in mild AD.

In moderate AD, CTLA-4 + CD8 + T cells are positively correlated with total tau (*p* = 0.0153), *p*- tau (*p* = 0.0277) and the number of CD28 + CD8 + T cells are negatively correlated with *p*-tau (*p* = 0.0336) and, like in mild AD (*p* = 0.0165), with Amyloid-β 1–40 (*p* = 0.0240). In severe AD, CTLA-4 + CD8 + T cells are positively correlated with *p*-tau (*p* = 0.0017; Table 2c).

### Correlation between co-stimulatory molecules and MMSE and Q Albumin

With the progression of dementia, the MMSE value as indicator for cognitive function declines. Correlation analyses between the MMSE values and the investigated T cell populations ruled out a positive correlation with CD28 + CD8 + T cells (*p* = 0.0466) and a negative correlation with CTLA-4 + CD4 + (*p* = 0.0003) in severe AD, illustrating that the decline of MMSE is associated with a decrease in CD28 expressing CD8 + T cells and an increase in CTLA-positive CD4 + T cells (Table 3a).

**Table 3:** Correlation between MMSE and Q Albumin and peripheral lymphocyte populations in AD and FTD

MMSE	Patients‘ group	
	Severe AD	
Cell population	CD28 + CD8 +	CTLA-4 + CD4 +
Pearson *r*	0.6092	− 0.8677
*P* value (two-tailed)	0.0466	0.0003
*P* value summary	*	***
*R* square	0.3711	0.7529

Q Albumin	Patients‘ group		
	FTD	moderate AD	FTD
Cell population	CD28 + CD4 +	CD28 + CD8 +	ICOS + CD8 +
Pearson *r*	0.8817	− 0.5891	− 0.9056
*P* value (two-tailed)	0.0480	0.0063	0.0130
*P* value summary	*	**	*
*R* square	0.7774	0.3470	0.8201

The correlation between MMSE and CD28 + CD8 + as well as CTLA-4 + CD4 + T cells in severe AD patients are shown in Table 3a

Table 3b depicts the correlation between Q Albumin and the frequency of CD28 + CD4 + as well as ICOS + CD8 + T cells in moderate AD and FTD

Q Albumin is a commonly used marker to determine the integrity of the B-CSF-B. In moderate AD, rising Q Albumin values as marker for a disturbed B-CSF-B are correlated with a falling frequency of CD28 + CD8 + T cells (*p* = 0.0063). In FTD, we detected a positive correlation between CD28 + CD4 + T cells (*p* = 0.0480) and a negative correlation between ICOS + CD8 + T cells (*p* = 0.0130) and Q Albumin (Table 3b).

## Discussion

The aim of our present study was to evaluate alterations in the expression of the co-stimulatory molecules CD28, CTLA-4 and ICOS in CD4 + and CD8 + T cells of AD, VD and FTD patients. In addition, we correlated these cell populations with dementia-associated changes such as decreasing MMSE values as marker for the progression of the cognitive decline but also with Q Albumin.

In AD we analyzed the stage-dependent changes in T cell co-stimulatory molecules in peripheral blood with disorder-specific modifications of p-tau, Amyloid-β 1–40, Amyloid-β 1–42 concentrations and the Amyloid-β ratio in CSF.

Previous studies found on the one hand a significant reduction of naïve CD28 + CD4 + T cells in AD patients [23, 24] and a decreased number of CD28 + CD3 + in VD [25], on the other hand an increase in CD28 + CD8 + and CD28 + CD4 + T cells were observed in AD patients [26, 27]. In our study, we detected a significant decreased frequency of CD28 + CD4 + T cells only in severe AD. CD28 is indispensable for the activation of naïve CD4 + and CD8 + T cells. The importance of this molecule for AD pathology is reflected by the strong negative correlation between the number of peripheral CD28 + CD8 + T cells and the concentration of tau proteins and Amyloid-β in CSF of AD patients and Q Albumin in moderate AD as well as a positive correlation with MMSE in severe AD. This illustrates that with progress of this disorder, when the concentrations of tau and Amyloid-β proteins and in particular also Q Albumin are altered and the MMSE value decreased, the number of CD28 + CD8 + T cells decreased as well. This might be explainable since despite CD28 is important for T cell activation and survival, several antigen-experienced T cells lose CD28 expression. These CD28- T cells are often described as terminally differentiated memory T cells [28]. With ongoing Alzheimer´s disorder, the chronic activation of T cells specific for the self-peptide Amyloid-β also continues which might also result in increasing numbers of CD28- clonally expanded T memory cells. Our data further support recent findings in peripheral and CSF CD8 + T cells from AD patients which show a shift towards memory T cells. These cells were shown to be clonal, antigen-experienced T cells that might patrol the intrathecal space of the brain which is affected by age-related neurodegeneration [29].

To our knowledge, the expression of ICOS had not been investigated in dementia until now. ICOS is another co-stimulatory molecule which was diminished in mild AD. ICOS supports a number of distinct processes during adaptive immune responses. It promotes T-dependent antibody responses [30] and drives antibody affinity maturation in the germinal center reaction [31, 32]. ICOS is involved in T helper cell polarization and might either enhance or dampen Th1 and Th2 inflammatory responses [33, 34]. In AD, and in particular in mild AD, the expression of ICOS is positively correlated with CD28 in CD4 + and in CD8 + T cells. Since ICOS is expressed only after activation, these cells might be activated or memory T cells. However, even much stronger is the correlation between ICOS and CTLA-4.

CTLA-4 is a homolog of CD28 and binds the same ligands, but with much higher affinity. In contrast to CD28, CTLA-4 is a potent inhibitory molecule. The expression of CTLA-4 is enhanced in AD (in particular moderate stage) and FTD by CD4 + T cells in VD by CD8 + T cells.

AD patients showed a peripheral increased pro-inflammation which is discussed to be involved in the pathogenesis of the disorder. We have shown an enhanced expression of VGF by T cells from AD patients compared to age-matched controls [35]. VGF contributes to the inflammatory response since blockade of VGF reduced the secretion of pro-inflammatory cytokines [36]. The expression of CTLA-4 might be induced to counter-regulate this immune imbalance.

Correlations between the peripheral expression of CD28, ICOS and CTLA-4 by CD4 + T cells and tau proteins and Amyloid-β in CSF were only detected in mild AD. Since both the numbers of CD4 + and CD8 + T cells were diminished in AD, one might speculate that CD4 + T cells are involved in early stages of AD, afterwards CD8 + T cells might be more important. Moreover, ICOS as well as CTLA-4 are also expressed by Treg cells. Treg cells are immunoregulatory lymphocytes which play an important role in modulating inflammation. Recently it was reported that ICOS signaling is important for the surface expression of CTLA-4, both molecules are important for the suppressive capacity of these cells [37]. In AD patients, a reduction of Treg cells was described [38] and a positive correlation between Treg in MCI and AD patients and MMSE scores was found [39]. It remains to be investigated whether the differential expression of both molecules by Treg cells contributes to the defective immune response seen in AD patients.

We found also in FTD patients an enhanced frequency of peripheral CTLA-4 + CD4 + T cells. Since enhanced CTLA-4 expression is associated with reduced immunoregulation and therefore inflammation, this might contribute to the inflammatory immune response widely observed in FTD patients in serum and CSF [9, 40, 41]. However, another group found a diminished number of CTLA-4-expressing CD4 + T cells in FTD [42].

Like in other forms of dementia, a dysfunction of the B-CSF-B, measured by Q Albumin, is also detected in FTD [43]. Impairment of the barrier has been attributed to oxidative stress and chronic inflammation. We found that with enhanced dysfunction of the B-CSF-B, the expression of ICOS by CD8 + T cells is diminished. This might be the consequence of an inflammation-mediated dysregulation between ICOS and CTLA-4 expression.

In VD patients, the number of CD8 + T cells was reduced while the frequency of CTLA-4 expressing CD8 + T cells was enhanced. CD28 and CTLA-4 compete for binding to the B7 molecules CD80 and CD86. Both molecules are expressed by professional antigen-presenting cells and provide co-stimulation following binding of a T cell [44, 45]. However, following ligation CTLA-4 exhibits much more power. This imbalance might contribute the pathology since it was shown that CD28 superagonist reduced brain damage after ischemic stroke in mice by amplification of Treg cells. The Treg cells attenuated the inflammatory response and improved outcome after experimental stroke [46]. Moreover, a recent study detected fewer Treg cells in VD [47]. This and the enhanced CTLA-4 expression further support the importance of inflammation in VD [11].

It has to be considered as well that AD is discussed as an autoimmune disorder [48, 49]. Taking into account that the expression of costimulatory molecules was found to be altered in autoimmune disorders and is discussed as potential target for treatment [50, 51], our findings might help to find new treatment opportunities for AD patients.

There are some limitations that have to be considered. The age of our control group is significantly different from that of the AD patients, who are older. However, no differences considering age were found between the non-demented volunteers and the other dementia groups as well as between AD and FTD and VD patients. Also, no significant differences in age within the patients suffering from the three stages of AD were found. Besides it is unexplained whether the alterations within the expression of costimulatory molecules, reflecting an altered immune system, contribute to the pathology of dementia or whether these changes are rather an epigenetic phenomenon. In this context it has to be considered that changes in the immune system, in particular (neuro-) inflammation, epigenetic and genetic pathways alter neurogenesis, which was found to be altered in AD raising the opportunity for therapeutic approaches, focusing on anti-inflammatory and pro-neurogenic interventions [52].

## Conclusions

The immune system in patients with several forms of dementia is differently modified. Disorder-specific alterations in the expression of co-stimulatory molecules might reflect the immune response after diverse stimuli. In AD, the stage-dependent changes in co-stimulatory molecules in T cells, combined with the correlation with AD-specific proteins, might be the consequence of a failed immune defense against these factors.

## Supplementary Information

Below is the link to the electronic supplementary material.

Supplementary file1 Supplementary table 1: Frequency of CD4+ T cells and CD8+ T cells in controls and patients with dementia. Suppl. Table 1a shows the mean percentage of peripheral CD4+ T cells, with SD and p value, suppl. Table 1b the mean percentage of peripheral CD8+ T cells from neuropsychiatric healthy controls and patients with MCI, AD (total, mild, moderate, severe) VD and FTD. * p compared with control group; n.s. = not significant (PDF 23 kb)

Supplementary file2 Supplementary Figure 1: Expression of CD28 and ICOS by CD4+ and CD8+ T cells in dementia. Shown are the frequencies of CD28-expressing CD4+ T cells (a) and CD8+ T cells (b) and the percentages of ICOS-expressing CD4+ T cells (c) and CD8+ T cells (d) (PDF 99 kb)
