# Supplementary figures and images for: Altered expression of costimulatory molecules in dementias
Source: Eur Arch Psychiatry Clin Neurosci. 2021 Aug 24;272(5):807–15. doi: 10.1007/s00406-021-01297-1 (PMC9279221; doi:10.1007/s00406-021-01297-1)

Supplementary Figure 1

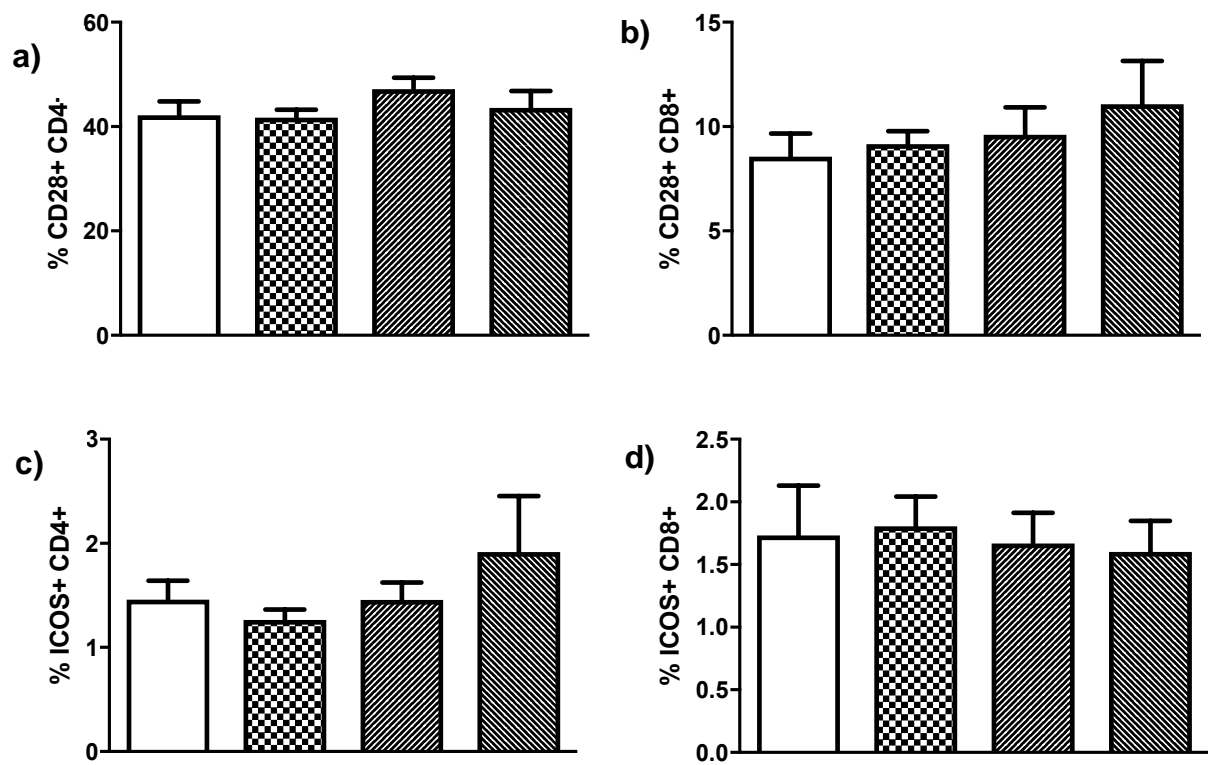

Supplement: Supplementary file 1 — Supplementary file1 Supplementary table 1: Frequency of CD4+ T cells and CD8+ T cells in controls and patients with dementia. Suppl. Table 1a shows the mean percentage of peripheral CD4+ T cells, with SD and p value, suppl. Table 1b the mean percentage of peripheral CD8+ T cells from neuropsychiatric healthy controls and patients with MCI, AD (total, mild, moderate, severe) VD and FTD. * p compared with control group; n.s. = not significant (PDF 23 kb) [file 406_2021_1297_MOESM1_ESM.pdf]
