# Supplementary material for: Altered expression of costimulatory molecules in dementias
Source: Eur Arch Psychiatry Clin Neurosci. 2021 Aug 24;272(5):807–15. doi: 10.1007/s00406-021-01297-1 (PMC9279221; doi:10.1007/s00406-021-01297-1)
Supplement: Supplementary file 2 — Supplementary file2 Supplementary Figure 1: Expression of CD28 and ICOS by CD4+ and CD8+ T cells in dementia. Shown are the frequencies of CD28-expressing CD4+ T cells (a) and CD8+ T cells (b) and the percentages of ICOS-expressing CD4+ T cells (c) and CD8+ T cells (d) (PDF 99 kb) [file 406_2021_1297_MOESM2_ESM.pdf]

Supplementary Table 1

a)

CD4+	control	MCI	AD				VD	FTD
			total	*mild*	*moderate*	*severe*		
mean (%)	20.2	18	12.6	12.4	13.1	11.9	12.7	13.5
SD	10.1	6	7.1	7.8	7.7	5.5	7.6	5.5
p		n.s.	0.0039	0.0225	0.0233	0.0141	0.0129	n.s.

b)

CD8+	control	MCI	AD				VD	FTD
			total	*mild*	*moderate*	*severe*		
mean (%)	13.3	9.8	8.3	7.7	7.9	9.7	8.3	8.1
SD	8.1	4	5.8	4.6	6.6	5.1	5.8	5.2
p		n.s.	0.0098	0.0373	0.0060	n.s.	0.0407	n.s.
